# Targeting the glutamine-arginine-proline metabolism axis in cancer

**DOI:** 10.1080/14756366.2024.2367129

**Published:** 2024-07-25

**Authors:** Di Wang, Jiang-jie Duan, Yu-feng Guo, Jun-jie Chen, Tian-qing Chen, Jun Wang, Shi-cang Yu

**Affiliations:** a Department of Stem Cell and Regenerative Medicine, Institute of Pathology and Southwest Cancer Center, Southwest Hospital, Third Military Medical University (Army Medical University), Chongqing, China; b International Joint Research Center for Precision Biotherapy, Ministry of Science and Technology, Chongqing, China; c Key Laboratory of Cancer Immunopathology, Ministry of Education, Chongqing, China; d Jin-feng Laboratory, Chongqing, China; e School of Pharmacy, Shanxi Medical University, Taiyuan, China

**Keywords:** Glutamine, proline, ALDH18A1, cancer, metabolism

## Abstract

Metabolic abnormalities are an important feature of tumours. The glutamine-arginine-proline axis is an important node of cancer metabolism and plays a major role in amino acid metabolism. This axis also acts as a scaffold for the synthesis of other nonessential amino acids and essential metabolites. In this paper, we briefly review (1) the glutamine addiction exhibited by tumour cells with accelerated glutamine transport and metabolism; (2) the methods regulating extracellular glutamine entry, intracellular glutamine synthesis and the fate of intracellular glutamine; (3) the glutamine, proline and arginine metabolic pathways and their interaction; and (4) the research progress in tumour therapy targeting the glutamine-arginine-proline metabolic system, with a focus on summarising the therapeutic research progress of strategies targeting of one of the key enzymes of this metabolic system, P5CS (ALDH18A1). This review provides a new basis for treatments targeting the metabolic characteristics of tumours.

## Glutamine addiction of cancer cells

Metabolic abnormalities are an important feature of cancers^1^. Rapidly proliferating cancer cells rely mainly on glutamine for survival and proliferation^2^. Glutamine is a nutrient that supports energy production and the synthesis of cellular biomass^3^. Additionally, this nutrient contributes to the supply of both carbon and nitrogen^3^. Glutamine is a widely recognised nutrient that is employed by cancer cells to increase proliferation and promote survival under conditions of metabolic stress^4^. As a result, glutamine serves as a rate-limiting factor for cell cycle progression, and in certain cellular contexts, the cell cycle cannot reach the S phase under conditions of glutamine depletion^5^.

Researchers have obtained a deeper understanding of the reprogramming of glutamine metabolism in cancer cells^6^. Glutamine provides energy for nucleotide, amino acid and lipid biosynthesis in growing cancer cells through the tricarboxylic acid (TCA) cycle and *via* its nitrogen and carbon skeleton^2^. As a result, most cancer cells are glutamine dependent and unable to survive without exogenous glutamine, a condition known as “glutamine addiction”^4^. Harry Eagle first demonstrated that the cervical cancer cell line HeLa requires 10 to 100 times more glutamine than other amino acids to maintain growth^7^. A variety of human cancer cells (such as pancreatic cancer, glioblastoma and small cell lung cancer cells) are sensitivity to glutamine starvation, which interferes with the metabolism of cancer cells and consequently leads to growth arrest and death^8^. Gliomas are glutamine-addicted cells. Increased expression of the glutamine transport protein SNAT3 in glioblastoma tissues has been reported to be a hallmark of malignant gliomas compared to low-grade gliomas and normal brain tissues^9^. Additionally, the glutamine transporter protein SLC1A5 (ASCT2) is also overexpressed in human glioblastoma cell lines and rat C6 glioma cells^10^. c-Myc in gliomas determines glutamine addiction through the activation of genes that encode proteins necessary for glutamine metabolism and uptake, such as SLC1A5 and glutaminase (GLS)^8^^,^^11^. Studies have identified molecular mechanisms linking upregulated GLS expression, glutaminolysis activation and the oncogene c-Myc in human prostate cancer cells and Burkitt’s lymphoma^12^. Hassanein et al. demonstrated the overexpression of SLC1A5 in adenocarcinoma, squamous cell carcinoma and neuroendocrine lung tumours^13^. Takeuchi et al. showed that SLC7A5 is expressed in non-small cell lung cancer (NSCLC) and that 2-aminobicyclo-(2,2,1)-heptane-2-carboxylic acid (BCH) inhibits SLC7A5 to reduce the viability of lung cancer cells^14^. In marked contrast to normal cells, lung cancer cells depend on glutamine due to the abnormal activation of oncogenes and the absence of tumour suppressors. These changes not only increase glutamine import *via* the upregulation expression of glutamine transporters but also promote the expression of metabolic enzymes involved in the metabolism of glutamine. For instance, a frequently disrupted route in lung cancer is the expansion of MYC functions *via* translocations and amplifications^15^. Abnormal glutamine metabolism was found in the serum metabolomic profile of natural killer T-cell lymphoma (NKTCL) patients, and SLC1A1 was found to be a key regulator of altered glutaminolysis. Ectopic expression of SLC1A1 accelerated cell proliferation and tumour development *in vitro* and *in vivo* by increasing cellular glutamine absorption, improving glutathione metabolic flux, and inducing glutamine addiction^16^. Glutamine addiction is a hallmark of clear cell renal cell carcinoma (ccRCC). Fu et al. revealed that ccRCC tumour cells with high cell-intrinsic glutamine metabolism activity had a worse survival rate than those with low activity. Cell-intrinsic glutamine metabolism in ccRCC tumours can enhance the immunosuppressive function of Treg cells through interleukin (IL)-23 while inhibiting the cytotoxicity of CD8 T cells. These findings support the use of IL-23 blockade as an effective therapeutic approach for treating glutamine-addicted ccRCC^17^.

However, there is significant variability in the response of cancer cell lines to glutamine deprivation, suggesting that not all cancer cells require exogenous glutamine for survival^18^. In breast cancer, basal-type cells rely on glutamine, while luminal-type cells tend to not rely on glutamine^19^. This difference in phenotype is linked to the luminal-specific expression of glutamine synthetase (GS), which is in turn regulated by a key luminal transcription factor called GATA3. Furthermore, GS inhibits the expression of GLS to shift the metabolic pathway towards glutamine synthesis in luminal breast cells and increase the possibility of glutamine symbiosis with basal breast cells^19^. Kim et al. discovered that tumour GLS expression is greater in TNBC than in other subtypes of breast cancer, and GLS is usually negative in HR-positive tumours^20^. A prior investigation indicated that glutamine is synthesised by glutamine synthetase in luminal-type breast cancer but not in basal-type breast cancer. Therefore, basal-type breast cancer or TNBC demonstrates elevated levels of GLS expression, rendering it more dependent on glutamine than other subtypes of breast cancer^18^^,^^19^. Furthermore, research has demonstrated that NSCLC cell lines exhibit differing degrees of reliance on glutamine, with 15 out of 38 lines displaying dependence on glutamine based on a reduction in growth rate of 70% or more in glutamine-free medium. Another 15 cell lines demonstrated an intermediate dependence on glutamine, with a reduction in growth rate ranging from 30 to 70%. Finally, 8 out of 38 lines demonstrated relative insensitivity to glutamine deficiency, with a 30% or lower decrease in cell growth^21^. B lymphoblastoid cell lines, including promyelocytic HL-60 cells, are highly reliant on glutamine for growth, while T-cell lines are able to proliferate in glutamine-free media. This dependence on glutamine is inversely related to the activity of GLS. Furthermore, GLS can be stimulated in glutamine-deficient media, especially glutamine-independent cells^22^.

## Sources of glutamine

In human blood, glutamine accounts for 20% of the total amino acids, whereas in muscle, glutamine accounts for 60% of all amino acids, and the muscle is an important site for endogenous glutamine production. The exogenous glutamine and muscle protein ingested through the digestive tract are under the action of GLS, and the endogenous glutamine produced is the main source of human glutamine^23^.

Cells can acquire glutamine *via* two methods. One is the transport of glutamine into cells from the microenvironment *via* SLC transporters, including members of the SLC1, SLC6, SLC7, SLC36, and SLC38 families^24^. Both the Na^+^/amino acid exchanger SLC1A5 and the unidirectional Na^+^/Cl^-^/amino acid isotropic transporter protein SLC6A14 are regulated by MYC and are often overexpressed in various types of cancer^24^. Furthermore, SLC7A5 (LAT1), which functions as a bidirectional transporter, facilitates the intracellular efflux of glutamine and the influx of leucine. Studies have shown that SLC1A5 mediates the entrance of glutamine into cells, which subsequently exits the cell due to the action of SLC7A5. This process is coupled with the entry of leucine into the cell, resulting in the synergistic activation of mammalian target of rapamycin (mTOR) signalling and thereby promoting cell growth and proliferation^25^. Notably, SLC3A2 is necessary for the formation of the SLC7A5-SLC7A11 heterodimer at the plasma membrane, which is also a target of MYC. In contrast, SLC6A14 is distinct from SLC1A5 and SLC7A5 because it transports substrates unidirectionally into cells, carries all essential amino acids, such as glutamine, and can couple SLC7A5 and SLC7A11^26^.

In the other method, cells synthesise glutamine through their own GS^27^. Mammals can synthesise glutamine in most tissues, and most free glutamine is synthesised and stored in skeletal muscle. In its physiological state, glutamine is generated by the combination of glutamate and ammonia through a reaction catalysed by GS. Intracellular glutamate can be synthesised in two ways: (1) glutamate can be synthesised from α-KG by glutamate dehydrogenase or multiple aminotransferases, and (2) glutamate can be synthesised from other amino acids (e.g. proline, arginine, and histidine)^3^. Most likely due to excessive glutamine uptake by cancer cells, the glutamine concentration in the microenvironment of cancer tissues is significantly lower than that in other tissues^28^. Thus, in glutamine-addicted cancer cells, some intrinsic oncogenic signalling pathways allow cancer cells to grow under conditions with a limited supply of glutamine^28^.

## Destination of glutamine

The functions of glutamine are as follows: (1) Glutamine acts as a nitrogen donor for purines and pyrimidines, thus providing amide nitrogen to allow for *de novo* synthesis of nucleotides, amino sugars, and NAD cofactors^28^. (2) Glutamine acts as a nitrogen donor for nonessential amino acids, and glutamine can be converted to proline and ornithine^28^. (3) Glutamine acts as a carbon donor. Endogenous and exogenous lipids are used by cells to form lipid membranes that are required for cell division. In xenograft models, inhibition of fatty acid synthesis prevents the growth of tumours, thus highlighting the importance of *de novo* lipid synthesis in tumour development. When hypoxia or mitochondrial dysfunction occurs, glutamine directly provides carbons for the synthesis of fatty acids and citrate^29^^,^^30^.

**Figure 1.**
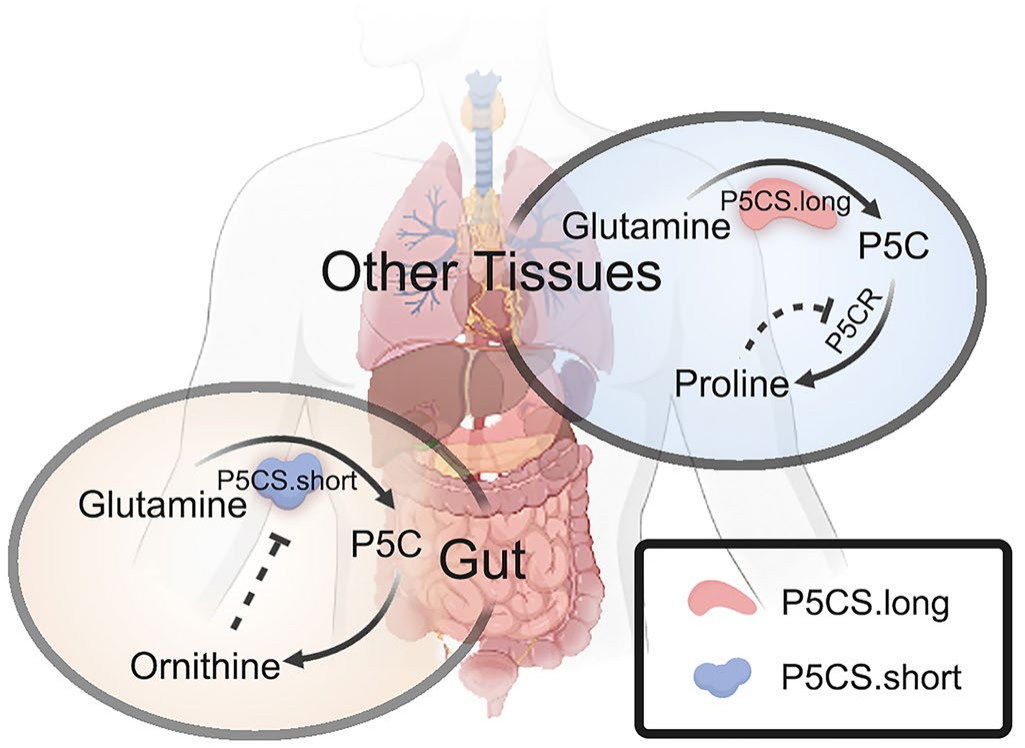
Functions of P5CS in different tissues. Mammalian P5CS undergoes alternative splicing to produce two isoforms. P5CS.short (the short isoform), which is inhibited by ornithine and converts glutamine to P5C, has high activity in the intestine. P5CS.long (the long isoform) has a broad tissue distribution, is insensitive to ornithine inhibition, and is involved in proline biosynthesis. This pathway is regulated by proline inhibition of P5CR. Abbreviations: P5C: pyrroline-5-carboxylic acid; P5CS: pyrroline-5-carboxylic acid synthase; P5CR: pyrroline-5-carboxylic acid reductase. The elements of the figure were downloaded for free from BioRender.com and then combined and drawn by the author.

Glutamine is transported intracellularly *via* the SLC1A5 transporter and can also be transported back to the extracellular space after being exchanged with leucine by the LAT1 (heterodimer of SLC7A5 and SLC3A2) anti-transporter^31^. The first step in glutaminolysis is the generation of glutamate through a reaction catalysed by cytoplasmic or mitochondrial glutaminases (GLS1 or GLS2). Glutamate has several fates. First, glutamate can be directly converted to GSH by the glutamate-cysteine ligase catalytic (GCLC) subunit^28^. Second, glutamate can be converted to α-KG by GLUD (GLUD1 or GLUD2) or transaminase and enter the tricarboxylic acid cycle^32^. Third, glutamate cyclisation produces proline. Glutamate is produced as γ-glutamyl phosphate (γ-GP) by the action of γ-glutamyl kinase (γ-GK) and later as γ-glutamyl semialdehyde (GSA) by the action of γ-GP reductase (γ-GPR). GSA is spontaneously cyclized to pyrroline-5-carboxylic acid (P5C), and P5C is reduced to proline by the action of pyrroline-5-carboxylic acid reductase (P5CR). Among these enzymes, γ-GK and γ-GPR are independent enzymes in bacteria and lower eukaryotes, whereas in higher organisms (e.g. humans and plants), γ-GK and γ-GPR are fused as single polypeptides to form a bifunctional enzyme, P5C synthase (P5CS), in which the γ-GK structural domain is the N-terminus and the γ-GPR structural domain is the C-terminus^33^. Fourth, glutamate generates arginine. In fact, glutamine, glutamate, proline and arginine are metabolic systems^34^. P5C generates ornithine under the action of ornithine delta transaminase (OAT), and ornithine then enters the urea cycle to generate arginine^35^. Remarkably, mammalian P5CS generates two isoforms, P5CS.short and P5CS.long, by selective splicing. P5CS.short is highly active in the intestine and is a key step in the catalytic arginine synthesis pathway, which can be inhibited by ornithine (Figure 1). P5CS.long is expressed in several types of tissues, is involved in glutamate-to-proline synthesis and is insensitive to ornithine inhibition (Figure 1); thus, this isoform allows cells to maintain proline synthesis when the ornithine levels are abnormally high^36^. Fifth, another major fate of glutamate derived from glutamine is extracellular excretion, in which glutamate can be exchanged with cystine *via* the xCT (isomer of SLC7A11 and SLC3A2) reverse transporter and then transported back to the extracellular space^31^.

## Glutamine-arginine-proline metabolism system

### Glutamine metabolism

High levels of glutamine in the blood provide an easily accessible supply of carbon and nitrogen for biosynthesis, energy, and cellular homeostasis; cancer cells may use glutamine to accelerate the growth of tumours^31^. Glutamine is assumed to play a pleiotropic role in cellular activities^31^. Glutamine may be directly incorporated into proteins to regulate protein trafficking and translation. For the production of nonessential amino acids and nucleotides, glutamine delivers carbon and nitrogen through catabolic reactions^37^. Thus, glutamine deficiency slows the development of some malignancies and may even cause cell death^38^. Additionally, some cancer cell lines use glutamine as a key nutrition source for oxidative metabolism^38^. Glutamine metabolism plays a crucial role in tumour progression by supporting mitochondrial oxidative phosphorylation, producing metabolic intermediates for the TCA cycle and for glutathione and NEAA synthesis and generating NADPH^39^. Recent studies have shown that pancreatic cancer cells use glutamine as a key fuel source for mitochondrial oxygen consumption. Additionally, the quantities of metabolites produced by glucose metabolism are impacted by variations in the expression of SLC1A5. Intriguingly, this study on increased glutamine metabolism in cancer cells also demonstrated that glutaminolysis might support metabolic reprogramming, which suggests that glutamine metabolism is essential for carcinogenesis and tumour growth^40^.

The excessive proliferative of cancer cells requires a steady supply of fuels such as glucose and glutamine^38^. Consequently, cancer cells regulate their metabolic pathways to meet their high nutrient demand. Dysregulated oncogenes are closely correlate with metabolic reprogramming, which increases glutamine consumption in cancer cells. Studies have attempted to elucidate the mechanism through which oncogenes alter metabolic pathways that promote cancer cell growth^41^. Notably, glutamine is essential for the survival of cancer cells with oncogenic MYC, K-Ras, and PIK3CA, and these cells use glutamine extensively for anabolic processes^42^. MYC is the oncogene that has most frequently been linked to increased glutamine metabolism^43^. MYC promotes the activity of the glutamine transporter SLC1A5, increases the expression of GLS, and activates the TCA cycle and glutathione synthesis under hypoxia^44^. In addition to mediating interactions with HER2 and the ER in breast cancer, MYC may also rewire glutamine metabolism once other oncogenic pathways are activated^31^. Through the overexpression of SLC1A5, glutamine absorption is increased in K-Ras-driven cells, similar to the results observed in MYC-driven cancer cells^45^. Low glutamine levels cause cell death, whereas K-Ras knockdown protects cells from this apoptosis. K-Ras sensitises cells to glutamine deprivation^46^. Taken together, these findings suggest that K-Ras-driven cancer cells exhibit increased glutamine metabolism and dysregulated cell growth, which promotes uncontrolled cell growth and facilitates glutamine acquisition and utilisation for cell growth^40^.

### Proline metabolism

**Figure 2.**
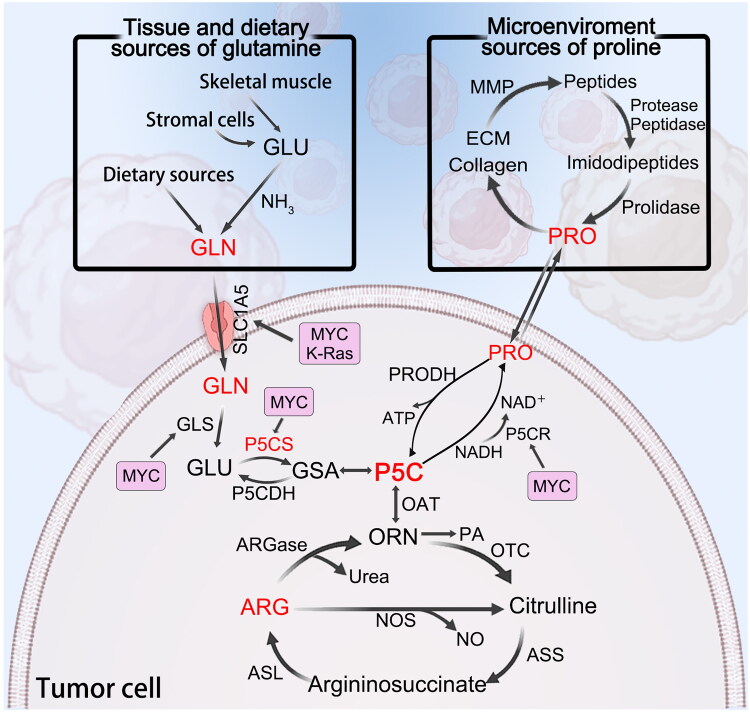
Interactions among glutamine-arginine-proline metabolism. P5C acts as a carbon skeleton and is an important intermediate metabolite in the interconversion of glutamine, arginine and proline. GSA and P5C are tautomers. Their interconversions are spontaneous. The sources of glutamine are shown on the top left, and the source of proline is shown on the top right. Glutamine enters the cell *via* SLC1A5 and can be converted to glutamate by glutaminase. P5CS is the first enzyme that converts glutamate to proline. Proline is converted to arginine *via* the P5C intermediate. Arginase then catalyses the forward conversion of arginine to ornithine, and OAT reversibly converts ornithine to P5C. Activation of the oncogenes K-Ras and MYC further increases SLC1A5 expression. MYC increases the gene expression of P5CS and PYCR, thereby increasing glutamine and proline uptake and metabolism rates. Abbreviations: ECM: extracellular matrix; GSA: gamma-glutamyl semialdehyde; P5C: pyrroline 5-carboxylate; GLU: glutamate; GLN: glutamine; PRO: proline; GLS: glutaminase; P5CS: P5C synthase; P5CDH: P5C dehydrogenase; P5CR: P5C reductase; PRODH: proline dehydrogenase; OAT: ornithine-δ-transaminase; ORN: ornithine; PA: polyamine; OTC: ornithine transcarbamylase; ASS: argininosuccinate synthase; ASL: argininosuccinate lyase; ARG: arginine; ARGases: arginase enzymes, including ARG1 and ARG2. The elements of the figure were downloaded for free from BioRender.com and then combined and drawn by the author.

Proline, a nonessential amino acid and the only imino acid that can be synthesised by the body, is produced in the cytoplasm and can be converted into a variety of amino acids and metabolic intermediates. Proline is thus indirectly involved in the tricarboxylic acid and the urea cycles. Proline can be converted to glutamate by proline dehydrogenase (PRODH)/POX and P5CDH on the inner mitochondrial membrane, whereas glutamate is converted to proline by P5CS on the inner mitochondrial membrane and P5CDH in the cytoplasm. P5CS in the inner mitochondrial membrane and P5CR in the cytoplasm are used to convert glutamate to proline. Among these enzymes, P5CS is the key enzyme involved in the synthesis of proline from glutamate in organisms. In addition, P5C acts as a carbon skeleton and is an important intermediate metabolite in the interconversion of glutamate and ornithine that links glutamate metabolism and the ornithine metabolism cycle^47^. Ornithine can also be converted to proline through the action of ornithine transaminase and P5CR^48^. The metabolic relationship between glutamate and proline mediated by P5CS in cancer cells is shown in Figure 2.

Proline, an important amino acid metabolite, is involved in a variety of cellular signalling pathways and ATP energy metabolism processes^49^; thus, proline affects many pathophysiological processes, such as cellular redox status; carbon and nitrogen metabolism; oxidative stress; and cell signalling, adaptation, and apoptosis, and plays an important role in tumorigenesis and progression^34^. Studies have shown that increasing proline level can increase the production of proteins, which are needed for cell proliferation. MYC and PI3K signalling, which promotes cell proliferation, increases the gene expression of P5CS and PYCR^50^. Increased expression of P5CS and PYCR1 is a poor prognostic factor for different types of cancers, which suggests that cancer cells require proline biosynthesis^51^. In addition, high expression of PYCR1 was detected in invasive breast ductal carcinoma, NSCLC and prostate cancer cells. Knockdown of PYCR1 inhibits cancer cell growth^52^. Richardson et al. reported that *de novo* proline synthesis is the major metabolic change in metastatic breast carcinoma cells^53^. Similarly, Denkert et al. reported a significant increase in the proline levels in invasive ovarian cancer tumours compared with that in ovarian junctional tumours^54^. De novo proline synthesis is also significantly increased in melanoma cells compared with melanocytes^55^. Vermeersch et al. demonstrated that the levels of metabolites in the proline and arginine metabolic pathways are significantly greater in ovarian cancer OVCAR-3 cells than in ovarian cancer stem cells. Similar to the finding that increased glycolytic content is an important carcinogenic phenotype, the reversal of proline accumulation in ovarian cancer stem cells may play a role in the stem cell-like phenotype^56^^,^^57^. Proline and ornithine (mainly proline) can induce the dose- and time-dependent differentiation of mouse embryonic stem cells (mESCs) into a new epiblast stem cell (EpiSC)-like state, and this state is induced by the breakdown of proline to P5C^58^. Proline is also considered a signalling molecule that controls stem cell behaviour, and physiological concentrations of proline are sufficient to convert ESCs into mesenchymal-like, highly motile, and invasive pluripotent stem cells, which then can induce *in vivo* metastasis^59^.

### Arginine metabolism

Arginine has multiple metabolic fates: arginine can be interchanged with glutamate and proline and serves as a precursor for the synthesis of proteins, nitric oxide, creatine, polyamines, agmatine, and urea^60^. Arginine is a conditionally essential amino acid that can be synthesised by the body itself in adult mammals under normal conditions, but under conditions such as starvation, inflammation, and small intestine or kidney dysfunction, the body does not synthesise a sufficient amount of arginine to meet metabolic requirements. Therefore, in adult mammals, arginine is a semiessential amino acid, whereas in newborns or preterm infants, this amino acid is essential^61^.

There are three main sources of free arginine in organisms: dietary protein, endogenous synthesis, and protein turnover^62^. Studies have shown that approximately 50% and 40% of the carbon for endogenous arginine synthesis originates from intestinal glutamine and proline, respectively^63^. P5CS is the first step in the *de novo* synthesis of arginine. In the small intestine, P5C is converted to ornithine *via* OAT and then to citrulline *via* OTC. Subsequently, citrulline is converted to arginine in the kidney *via* the urea cycle cytoplasmic enzymes argininosuccinate synthetase (ASS) and argininosuccinate lyase (ASL). In addition, under starvation conditions, a portion of the ornithine synthesised by intestinal P5CS and OAT is transferred to the liver to replenish the urea cycle^64^. Studies have shown that in the neonatal gut, most glutamine-derived P5C is converted to ornithine rather than proline, and ornithine is subsequently used for the efficient synthesis of citrulline and arginine^65^.

In contrast to the finding that a single enzyme synthesises arginine, five enzymes use arginine as a substrate^66^. (1) Arginyl-tRNA synthetase (ArgRS) uses arginine as a substrate but is not considered a metabolic enzyme, and charged tRNAArg is not only necessary for protein synthesis but also plays a role in protein degradation^61^. (2) Arginine decarboxylase (ADC) catalyses the conversion of arginine to CO_2_ and agmatine^67^. (3) Arginine-glycine amidinotransferase (AGAT) is involved in the first step in creatine synthesis^68^. (4) There are two ARG isozymes. Arginine can be hydrolysed by cytoplasmic ARG1 and mitochondrial ARG2 to produce urea and ornithine, which in turn can serve as precursors for the synthesis of polyamines, proline, or glutamate^69^. (5) Nitric oxide synthases (NOSs) catalyse the oxidation of arginine to NO and citrulline (Figure 2)^69^.

ASS1 is recognised as the rate-limiting enzyme in arginine synthesis, and in many tumours, including hepatocellular carcinoma, melanoma, prostate cancer, and renal cancer, ASS1 expression is downregulated or even absent, which prevents these tumours from synthesising arginine and thus makes them dependent on exogenous arginine. These tumours are thus sensitive to arginine deprivation therapies and are termed arginine-nutrient-deficient tumours^70^. Arginine deprivation is emerging as a promising new clinical treatment strategy for metabolism-based cancer therapy^71^. In tumour cells that do not express ASS1, promoting the degradation of extracellular arginine triggers apoptosis^72^. Arginine deficiency leads to GCN2-dependent cycle arrest in HCC cells, and inhibition of GCN2 in arginine-deficient HCC cells enhances the efficacy of senescent compounds and promotes cellular senescence^73^. Arginine promotes the transition of activated T cells from glycolysis to oxidative phosphorylation. Additionally, arginine promotes the growth of more viable central memory-like cells that exhibit antitumour effects in mouse models^74^. Abnormal upregulation of ARG1 and ARG2 expression has been observed in a range of cancers. ARG1 overexpression increases ERK and AKT phosphorylation in neuroblastoma (NB), which promotes cell growth^75^. ARG2 expression is markedly greater in malignant thyroid tumours than in normal tissues, and the suppression of ARG2 decreases AKT expression while increasing tumour cell apoptosis^76^. Phosphorylated STAT3 can directly bind to the ARG1 promoter region of myeloid-derived suppressor cells (MDSCs) in head and neck squamous cell carcinoma (HNSCC) to stimulate transcription and enhance the immunosuppressive properties of MDSCs^77^. ARG2 stimulates the metastasis of tumours in melanoma *via* the H2O2-STAT3 pathway^78^.

### Interactions among glutamine-arginine-proline metabolism

Glutamine, arginine, and proline form a metabolic system with P5C as the central metabolite^34^. The interconnected networks of this metabolic system are shown in Figure 2. Proline is converted to arginine *via* the P5C intermediate. Arginase (ARG) then catalyses the forward conversion of arginine to ornithine, and OAT reversibly converts ornithine to P5C^79^. We were intrigued by the peculiar aspect of amino acid metabolism in which P5C is not only the precursor for proline synthesis but also the direct result of proline catabolism^34^. As a result, P5CS is the first enzyme that converts glutamate to proline^49^, and the glutamate-P5CS pathway accounts for most proline production in the human body^80^. Notably, proline cannot be metabolised by common amino acid enzymes (i.e. racemases, transaminases, and decarboxylases) because it contains an amino group in the pyrrolidine ring. Instead, a specific family of enzymes with their own subcellular localisation and regulatory mechanisms has evolved, and these enzymes mediate the interconversion of glutamine, proline, and arginine^34^^,^^81^. A crucial step in the production of proline, ornithine, and arginine is the catalysis of glutamate by P5CS to produce P5C^64^. P5C is the precursor of proline; therefore, after a significant loop, this process returns to the beginning, i.e. proline. The “glutamine-arginine-proline metabolic axis” is thus formed by these metabolic pathways^79^.

The glutamine-arginine-proline axis is a crucial aspect of cancer metabolism and controls a significant proportion of amino acid metabolism. The production of other nonessential amino acids and necessary metabolites also relies on this axis as a framework^79^. Under various nutritional stress conditions, glutamine, arginine, and proline interconversions are significant^34^. Although some studies in adult humans have shown that enteral glutamine provides approximately 50% of the available carbon for arginine synthesis and that proline provides approximately 40% of the available carbon^82^^,^^83^, other research groups have shown that glutamine contributes more substantially to endogenous arginine synthesis in humans^63^. In addition, the formation of alpha KG from glutamate derived from either proline or arginine may play an anaplerotic role in the TCA cycle^34^. P5C acts as a carrier or transfer mechanism for the pyridine nucleotide-dependent oxidising, regardless of whether it is generated from arginine by ornithine, glutamate by P5CS, or proline by POX/PRODH^34^. In addition, the production of polyamines is derived from arginine^79^. An indication that glutamine may be converted to arginine for polyamine production is that the beneficial effects of glutamine deprivation on T-cell activation are partially reversed by polyamine supplementation^84^. These findings reinforce the already significant role of glutamine in cancer growth and proliferation and highlight the importance of the glutamine-arginine-proline metabolic pathway in cancer progression and treatment.

## Advances in cancer therapy targeting the glutamine-arginine-proline metabolism axis

The important role of the glutamine-arginine-proline metabolic pathway in the growth of cancer cells provides opportunities for cancer therapy targeting metabolism. To date, several studies have investigated the targeting of important proteins and enzymes in the glutamine, arginine and proline metabolic pathways to interfere with glutamine-arginine-proline metabolism^85^. (1) Glutamine metabolism: inhibitors such as glutamine transporters, GLS and transaminase have shown certain effects in preclinical and clinical treatment trials in patients at different stages of cancer. The current strategy for developing inhibitors is based on maintaining the level of glutamine metabolism needed for normal tissue physiology while impairing glutamine addiction in cancer cells^86^. The first step is to inhibit glutamine uptake, which can be depleted from the extracellular space by using L-asparaginase^31^^,^^87^. Alternatively, glutamine transport can be dampened by inhibiting ASCT2 uptake *via* GPNA and V-9302. Second, the conversion of glutamine to glutamate can be inhibited by inhibiting GLS. The relevant inhibitors include DON, azaserine, acivicin, PTES, CB-839, and the small-molecule inhibitor 968, which can block GLS activity and glutaminolysis. Among these agents, DON, azaserine, and acivicin have been discontinued as early antagonists of glutamine metabolites due to excessive side effects, such as dose-limiting neurotoxicity, gastrointestinal toxicity (nausea and vomiting), and bone marrow suppression, even though they all exerted potent antitumour effects and exhibited clinical activity^87^. In light of the clinical potential of DON, several DON prodrugs (such as JHU-083 and DRP-104) have been developed to effectively decrease gastrointestinal toxicity^88^. JHU-083 is activated *in vivo* through the proteolytic hydrolysis of enriched enzymes (such as cathepsin-L), leading to the release of active DON. As a consequence, JHU-083 exhibits enhanced oral bioavailability and reduced toxicity^89^. The stability of DRP-104 in human plasma and gastrointestinal tissues, as well as the selective targeting of DON to tumours, have demonstrated its potential as an effective antitumour drug in various preclinical models without significant toxicity. Consequently, this chemical has been chosen for clinical trials aimed at combating NSCLC and other solid tumours^90^. BPTES and its derivative CB-839 inhibit the transition of GLS from dimer to tetramer, and the small-molecule inhibitor 968 inhibits the activity of Rho GTPases and thereby inactivates GLS^31^. Among these agents, CB-839 has entered phase II clinical trials for the treatment of PIK3CA-mutant colorectal cancer (NCT02861300), NSCLC (NCT03965845) and triple-negative breast cancer (NCT03057600). In addition, there are many novel GLS inhibitors. However, to date, only CB-839 has entered clinical trials for the treatment of advanced solid tumours and haematologic malignancies^91^. (2) Arginine metabolism: ASS1 is recognised as the rate-limiting enzyme in arginine synthesis, and in many cancers, including hepatocellular carcinoma, melanoma, prostate cancer, and renal cancer, ASS1 expression is downregulated or even absent, thus preventing cells from synthesising arginine and making them dependent on exogenous arginine. These cancers are thus sensitive to arginine deprivation therapies and are termed arginine-nutrient-deficient cancers^92^. Arginine deprivation is emerging as a promising new clinical treatment strategy for metabolism-based cancer therapy^93^. The antitumour activity of ADI-PEG20, an inhibitor targeting ADI, has been further demonstrated in pancreatic, prostate, small cell lung, and breast cancers^71^. Studies have shown that the antitumour activity of ADI-PEG20 is correlated with ASS1 deficiency in tumours, and its therapeutic effect may be eliminated when ASS1 is restored^70^. Phase III clinical trials of ADI-PEG20 for the treatment of advanced or metastatic smooth muscle sarcoma (NCT05712694) and phase III clinical trials for the treatment of patients with advanced liver cancer (NCT05712694) are currently underway. (3) Proline metabolism: the discovery of inhibitors targeting PYCR is at an early stage^94^. A remaining unresolved challenge is the high-resolution structure of human PRODH and PYCR. Screening for human PRODH inhibitors will be more challenging due to the lack of a convenient method for generating recombinant enzymes^33^. Tanner et al. identified five small-molecule inhibitors of PYCR through a small-scale focused screening of proline analogues using X-ray crystallography. Of these, NELP was the most potent inhibitor and achieved its inhibitory effect by occupying the binding site of P5C^94^^,^^95^ (Table 1).

**Table 1.** Pharmacological strategies targeting the glutamine-arginine-proline metabolic system in cancer.

Class	Mechanism	Drug	Cancer type	Clinical phase	Refs.
Glutamine metabolism	ASCT2 inhibitors	GPNA	Breast cancer	Preclinical phase	28
		V-9302	Breast cancer	Preclinical phase	126
	Glutamine mimic	DON	Breast cancer, colorectal cancer, lung cancer, acute leukaemia	Phase II clinical trial (terminated)	31,87,127
		Azaserine		Phase II clinical trial (terminated)	
		Acivicin		Phase II clinical trial (terminated)	
	DON prodrugs	JHU-083	Medulloblastoma	Phase not applicable	89,128
		DRP-104	NSCLC, prostate cancer, other solid tumours	Phase II clinical trial	88,90
	GLS inhibitors	968	Breast cancer	Preclinical phase	39,85,124
		BPTES	Breast cancer, liver cancer	Preclinical phase	
		CB-839	NSCLC, lymphoid, myeloid malignancies and triple-negative breast cancer	Phase II clinical trial	
Arginine metabolism	Arginine deprivation	ADI-PEG20	Pancreatic cancer, prostate cancer, lymphoma	Phase III clinical trial	62
	ARG inhibitors	CB-1158	Advanced/metastatic solid tumours	Phase II clinical trial	129
		nor-NOHA	Breast cancer, colorectal cancer, hepatocellular carcinoma	Phase not applicable	69
		OATD-02	Glioblastomas, ovarian cancer	Phase I clinical trial	69
Proline metabolism	Proline analogue	NFLP	Breast cancer	Phase not applicable	94,95

**Figure 3.**
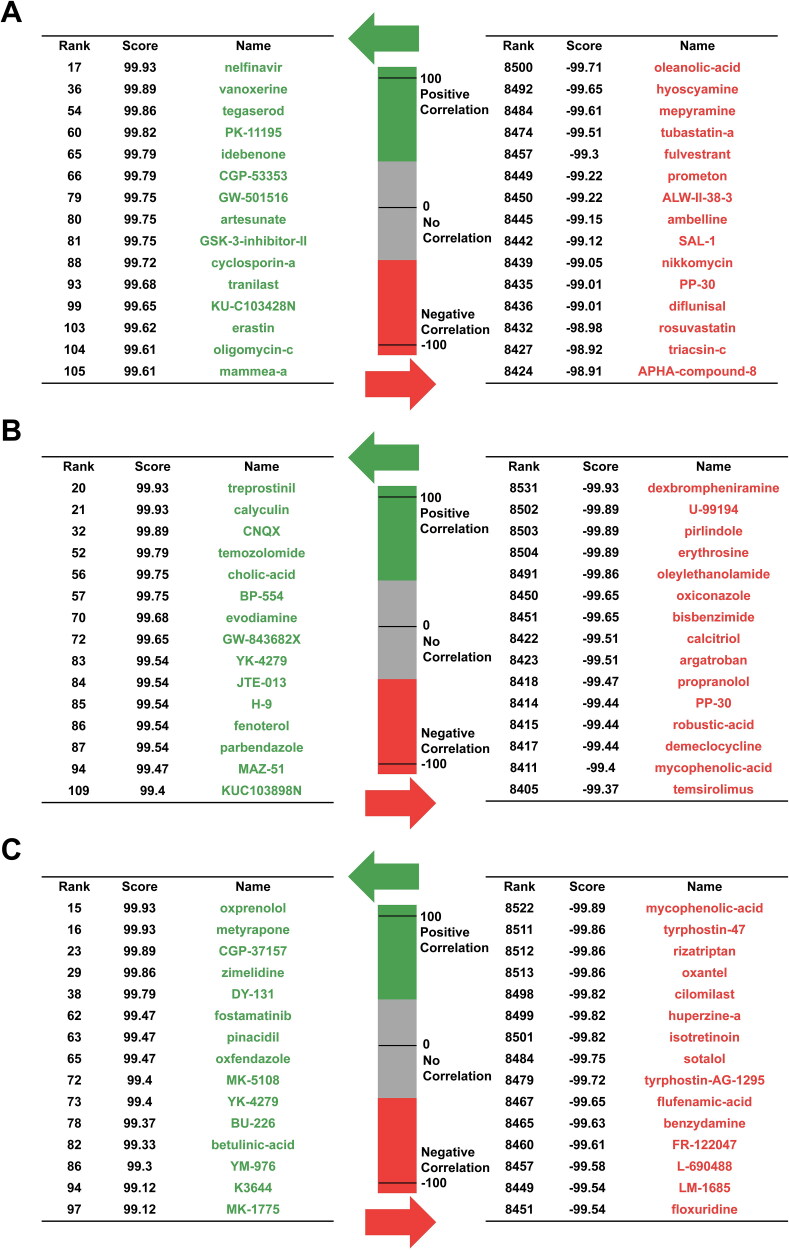
CMap analysis was performed to identify potential therapeutic drugs that target glutamine, arginine and proline metabolism. (A) CMap analysis was performed to identify potential therapeutic drugs that target glutamine metabolism. (B) CMap analysis was performed to identify potential therapeutic drugs that target arginine metabolism. (C) CMap analysis was performed to identify potential therapeutic drugs that target proline metabolism. Potential therapeutic drugs were analysed using Connectivity Map (https://clue.io)^96^.

As Connectivity Map (CMap) can be used to discover the mechanism of action of small molecules, functionally annotate genetic variants of disease genes and inform clinical trials, correlating the main data with that from other clinical databases would be fascinating^96^^,^^97^. We enriched the genes related to glutamine metabolism, arginine metabolism and proline metabolism by KEGG pathway analysis and used “Qurey” in the CMap online database to obtain the information about interactions between genes and drugs. Unfortunately, the corresponding small molecules listed in Table 1 were not found (Figure 3).

As mentioned above, various strategies and small-molecule inhibitors have been successively developed to target all aspects of glutamine-arginine-proline metabolism in cancer cells. P5CS, encoded by ALDH18A1, is the key enzyme that catalyses the conversion of glutamate to proline, ornithine and arginine^65^. Changes in ALDH18A1 undoubtedly cause alterations in the glutamate-proline-arginine metabolic system, and these alterations affect the processes of tumorigenesis and progression. Therefore, a further review of the clinicopathological characteristics, evolution, clinical significance, and drug development prospects of ALDH18A1 and cancers is provided below^98^.

## ALDH18A1 and cancers

### ALDH18A1 protein structure

**Figure 4.**
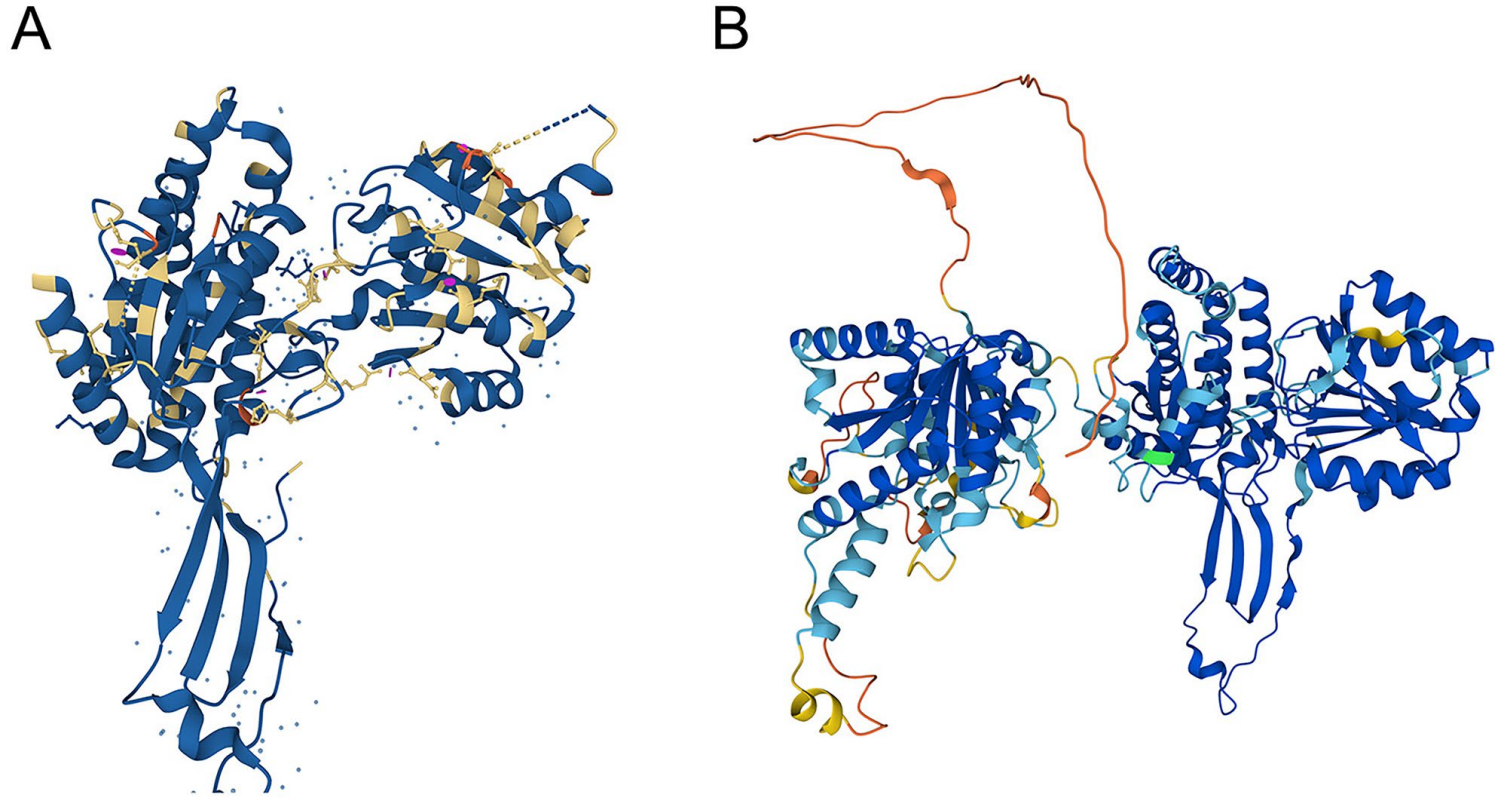
Crystal structure of human P5CS. (A) Crystal structure of human P5CS (PDB ID 2H5G) at amino acid positions 362–795 from the PDB database. (B) Predicted crystal structure of human P5CS (AF-P54886-F1) at amino acid positions 1–795 from the AlphaFold protein structure database. The crystal structure of human P5CS is available for free consultation and download on the Uniprot online platform (https://www.uniprot.org) ^132^.

ALDH18A1 is located on chromosome 10 (10q24.3), contains 19 exons and is approximately 50 kb in length. P5CS contains two enzymatic structural domains, γ-GK (amino acids 1 to 361) and γ-GPR (amino acids 362 to 795): the former contains 64 amino acid residues in the mitochondrial targeting sequence that mediate the phosphorylation of glutamate to form γ-GP, and the latter converts γ-GP, an unstable intermediate reaction product, into P5C and its isomer GSA. The crystal structure of γ-GPR has been resolved (PDB ID 2H5G) (Figure 4(A)), and the crystal structure of the GK portion of P5CS remains unresolved but was predicted by the AlphaFold protein structure database (Figure 4(B))^99^.

### Expression and significance of ALDH18A1 in cancers

As an important aldehyde dehydrogenase isoform ALDH18A1 exhibits the highest activity in the small intestine and is expressed in the colon, thymus and brain^65^. ALDH18A1 is involved in the generation of a variety of epigenetically regulated intermediates in cancer, such as α-KG^100^, NAD(P)^+^^57^, and SAM^101^, and plays an important role in metabolic reprogramming and epigenetic regulation of the effects of interconnections in cancer.

In P493 and PC9 cells, ALDH18A1 affects NAD^+^/NADH to regulate the glycolytic pathway and thereby regulates proliferation by altering cellular energy metabolism; furthermore, ALDH18A1 affects NADP^+^/NADPH to regulate the pentose phosphate pathway and proliferation *via* nucleotide metabolism^57^. P5C can be converted to proline in the mitochondria or cytoplasm *via* PYCR using NADPH or NADH as a cofactor for conversion to proline. Thus, the interconversion of P5C and proline leads to the recirculation of cellular NAD(P)H to NAD(P)^+^^102^. Consistent with recent studies, Ding et al. also reported that ALDH18A1 can influence the glycolysis and pentose phosphate pathways in hepatocellular carcinoma cells through the production of ADP^+^, which is essential for nucleotide biosynthesis and the production of acetyl coenzyme A^103^. Thus, ALDH18A1 promotes hepatocellular carcinoma cell proliferation, and inhibition of ALDH18A1 may be a novel therapeutic strategy for hepatocellular carcinoma^103^.

**Figure 5.**
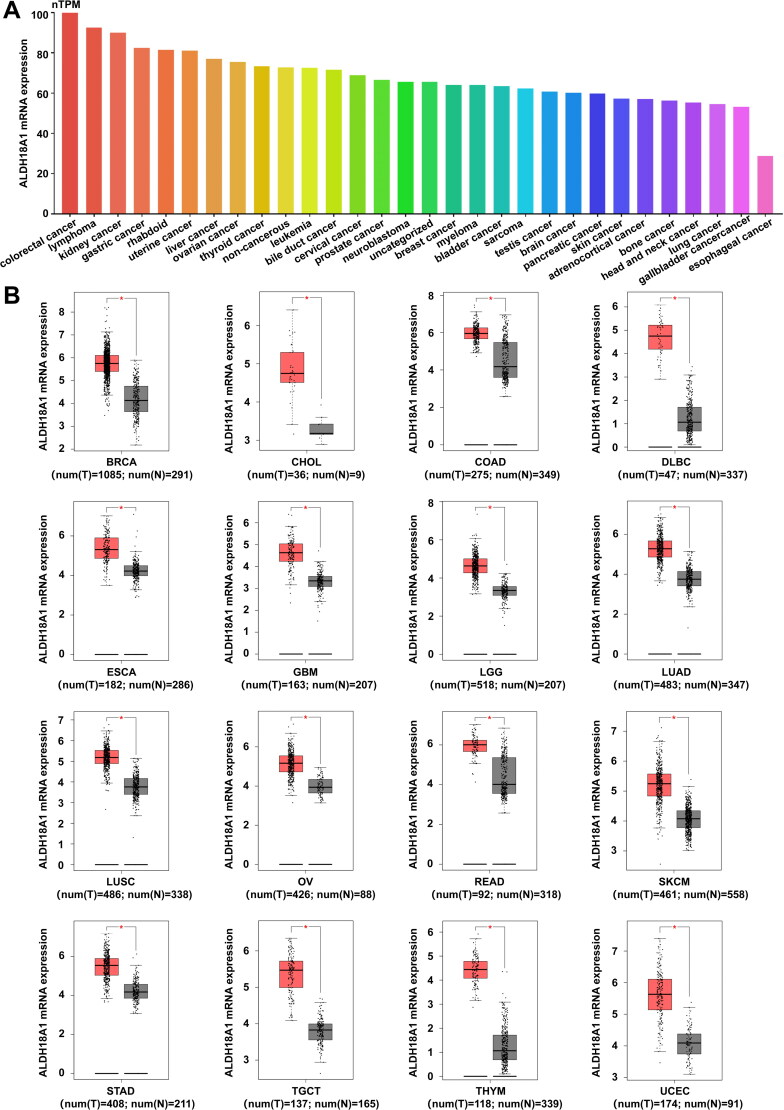
ALDH18A1 mRNA expression in patients with various types of cancer. (A) ALDH18A1 mRNA expression data are expressed as normalised transcript per million (nTPM) values in 1206 cancer cell lines from the Human Protein Atlas (HPA) database. The data are based on HPA version 23.0 and Ensembl version 109. (B) Transcript expression of ALDH18A1 in clinical tumour samples and normal tissues in the GEPIA database. The red box plots show tumour expression, while the grey colours represent normal tissues. ALDH18A1 mRNA expression was analysed using the free public databases Human Protein Atlas (HPA, http://www.proteinatlas.org)^133^ and Gene Expression Profiling Interactive Analysis (GEPIA, http://gepia.cancer-pku.cn/)^110^.

In addition, hypoxia or ETC inhibition induces reductive glutamine metabolism, in which glutamine is converted primarily to acetyl coenzyme A for fatty acid biosynthesis^37^. Cancer cells often survive under hypoxic conditions and undergo metabolic reprogramming. Furthermore, the knockdown or inhibition of ALDH18A1 in luminal B breast cancer may contribute to the therapeutic effects resulting from targeting this enzyme. Furthermore, high protein expression of glutamine-prolinases (GLS, ALDH18A1 and P5CR) is associated with high c-Myc protein expression in only luminal B breast cancer, suggesting that c-Myc is the driving force behind the metabolic state of the breast cancer subclass^104^. Proline biosynthesis is significantly enhanced in melanoma cells compared with normal melanocytes. The inhibition of ALDH18A1 can inhibit cancer growth by 60% to 99% and reduce the viability of melanoma cells by 90%, whereas exogenous supplementation with proline can reverse this effect^105^. In addition, knockdown of MYC significantly reduces proline and hydroxyproline production in hepatocellular carcinoma cells under hypoxic conditions, indicating that the glutamine metabolic axis *via* MYC/GLS and MYC/ALDH18A1 is essential for proline synthesis under hypoxic conditions^106^. Furthermore, Fang et al. reported that ALDH18A1 and PYCR1 are frequently overexpressed in NB cells and human cancers and are associated with poor patient prognosis, whereas the deletion of ALDH18A1 or PYCR1 decreases proline levels and reduces tumorigenesis^49^^,^^105^. Fang et al. reported that the expression levels of ALDH18A1 or PYCR1 are associated with poor prognosis in NB patients^107^. Consistent with the results of this study, our previous study also demonstrated that the expression of ALDH18A1 was significantly associated with overall survival of NB patients. Administration of the ALDH18A1-specific inhibitor YG1702 inhibits N-MYC expression and attenuates NB cell growth^108^. In conclusion, ALDH18A1 may be a potential drug target for cancer therapy (Table 2). To further verify the expression of ALDH18A1 in different tumour cell lines, we downloaded cell line data from the Human Protein Atlas (HPA) database^109^. The HPA was used to summarise the transcriptional level of each gene in 1206 cell lines. The analysed cell lines were grouped according to cancer type (Figure 5(A)). In addition, we further verified the expression of ALDH18A1 mRNA in different tumour tissues and normal tissues using the Gene Expression Profiling Interactive Analysis (GEPIA) online database^110^^,^^111^, and the results showed that the expression of ALDH18A1 was upregulated in 16 types of tumour tissues compared to that in normal tissues (Figure 5(B)).

**Table 2.** ALDH18A1 is highly expressed in diverse types of cancer.

No.	Cancer type	Conclusion	Refs.
1	Burkitt lymphoma, prostate cancer	MYC regulates proline biosynthesis and proliferation.	130
2	Burkitt lymphoma, NSCLC	ALDH18A1 has the ability to regulate proliferation through both the glycolytic and pentose phosphate pathways.	57
3	Hepatocellular carcinoma	ALDH18A1 is capable of regulating cell proliferation in hepatocellular carcinoma by modifying the pentose phosphate pathway.	103
4	Melanoma	ALDH18A1 affects proline metabolism and thus regulates protein synthesis through GCN2 and eIF2a and thereby melanoma cell proliferation.	105
5	Cervical cancer, breast cancer	ALDH18A1 regulates glutamate-proline metabolism and changes the intracellular acetyl-CoA levels, which results in impacts on lipid metabolism.	131
6	Hepatocellular carcinoma	ALDH18A1 affects the stability of HIF1α by regulating the level of proline metabolism and thereby regulates the proliferation of hepatocellular carcinoma cells.	106
7	Neuroblastoma	ALDH18A1 regulates neuroblastoma cell proliferation and invasiveness by modulating the proline metabolism levels.	107
8	Breast cancer	Higher ALDH18A1 expression is observed in ER^+^HER2^high^ (luminal B) breast cancers compared with ER^+^HER2^low^ (luminal A) breast cancers and is strongly associated with poor patient prognosis.	104

### Anticancer therapy targeting ALDH18A1

ALDH18A1 is highly expressed in a range of tumours, including NB^108^, hepatocellular carcinoma^103^ and luminal B breast cancer^104^. Because it is a crucial enzyme in the glutamine-arginine-proline metabolic system, targeting ALDH18A1 *via* anticancer therapies could create novel opportunities for cancer intervention. To identify specific small-molecule inhibitors of ALDH18A1, structure-based computerised virtual screening, which is a very effective method for high-throughput screening of drugs, was utilised. Structure-based virtual screening includes various sequential computational stages, target and database preparation, docking and postdocking analysis, and prioritisation of test compounds^112^. Among these steps, conformational changes due to interactions with ligands and structural resolution are important details to be considered when selecting the most suitable structure. However, the current algorithms cannot estimate the absolute energy associated with intermolecular interactions with satisfactory accuracy. The appropriate evaluation of solvent effects, entropic effects and receptor flexibility are the main challenges that need attention.

The P5CS protein encoded by ALDH18A1 is known to contain two enzymatic structures, the G5PR and G5K structural domains. The crystal structure of the G5K portion of P5CS has not been resolved. Therefore, we used molecular docking to screen for P5CS-specific small-molecule inhibitors based on the structure of G5PR and ultimately selected the compound with the highest docking score to P5CS, namely, YG1702^108^. Furthermore, we verified the specific binding between YG1702 and the ALDH18A1 recombinant protein using isothermal titration calorimetry and found that YG1702 has a high affinity for ALDH18A1 and may affect its enzymatic activity^108^. Because the partial structure of G5K, another enzymatically active structural domain of human P5CS, has not been resolved, screening of inhibitors with enzymatic activity of this structural domain is difficult. However, the crystal model of the catalytic structural domain of *Escherichia coli* G5K has been successfully resolved, and ALDH18A1 is highly conserved in a variety of organisms^113^. Proteins with relatively high sequence and functional conservation are more desirable target molecules for drug design. If there is a mutation of a single amino acid residue within the active site of the protein (point mutation) that may affect ligand binding, the drug molecule targeting the mutated protein will not exhibit the desired effect due to the high likelihood of mutations in the sequence of the structural domain^114^. In addition, an important issue in molecular docking is finding the best binding site between the substrate and the target enzyme. Due to the complexity and variability of the tertiary structure of proteins, identifying a suitable region where the ligand can bind easily is the basis of molecular docking methods. The results of molecular docking are extremely sensitive to protein conformation^115^. Therefore, the structural conservation of proteins is highly convenient for drug development^116^.

In addition, there is a need to investigate the possible side effects of targeted therapy for ALDH18A1, which is a key enzyme involved in the glutamate-arginine-proline metabolic system and therefore essential for normal tissue function. Defects in ALDH18A1 may lead to congenital disorders such as hypoprolinemia, hypoarginemia, neurodegeneration, and connective tissue abnormalities^117^. Determining whether these phenotypes occur when targeting ALDH18A1 is very important, and the possible side effects can be mitigated when cancer treatments are targeted. In addition, drugs targeting ALDH18A1 should not disrupt the function of other ALDH family members that may be necessary for normal physiological processes^105^, such as ALDH1As, an important subfamily of ALDH that includes 3 members: ALDH1A1, ALDH1A2 and ALDH1A3. ALDH1As and ALDH8A1 jointly catalyse the second step of RA synthesis, namely, the oxidation of all-trans or cis-retinal to RA, and are important regulatory genes involved in RA synthesis^118^; RA synthesis is essential for the normal growth, development and maintenance of adult vertebrate epithelial cells^119^. ALDH2, as a nitrate reductase, is the main enzyme necessary to activate nitroglycerine and used to treat angina and heart failure^120^. ALDH3A1 is considered a corneal crystallin, is one of the most abundantly expressed proteins in mammalian corneal epithelial cells, and accounts for more than 50% of the total water-soluble protein fraction, and corneal ALDH3A1 protects the cornea and underlying lens from UV-induced oxidative stress^121^.

## Prospects

Metabolic abnormalities are one of the hallmarks of cancer. Metabolic reprogramming promotes cancer cell survival, proliferation, angiogenesis and metastasis. An increasing number of in-depth studies on cancer metabolism have shown a strong link between amino acid metabolism and cancer behaviour^122^. Glutamine is the most abundant amino acid in plasma, is used to replenish the TCA cycle and provides carbon and nitrogen for the synthesis of nucleotides, amino acids and glutathione. Notably, glutamine, arginine and proline metabolism play important role in epigenetic regulation by altering the expression of tumour oncogenes and tumour suppressor genes and the cancer cell phenotype^123^^,^^124^. Considering the broad opportunities for targeted metabolic therapies for cancer, a key question is whether researchers have exhausted all metabolic hubs as targets, despite the successive development of several strategies and small-molecule inhibitors targeting various aspects of glutamine-arginine-proline metabolism in tumour cells^31^. In particular, changes in ALDH18A1, a key enzyme in the glutamine-arginine-proline metabolic system, undoubtedly affect the processes of tumorigenesis and progression^105^. ALDH18A1 not only regulates proline metabolism in cancer cells such as melanoma, NB and hepatocellular carcinoma cells but also controls the pentose phosphate pathway by altering the NADP^+^/NADPH ratio, which in turn regulates cell proliferation through nucleotide metabolism in cancer cells such as lymphoma, NSCLC and hepatocellular carcinoma cells^57^^,^^103^. Therefore, further exploration of the function of ALDH18A1 in cancers has become increasingly relevant.

We previously used a molecular docking approach to screen for specific ALDH18A1 inhibitors. Among these compounds, YG1702 exhibited low cytotoxicity in normal human brain glial (HEB) cells and human colonic mucosal epithelial NCM460 cells but high cytotoxicity in MYCN-amplified NB cells. Isothermal titration calorimetry (ITC) showed high affinity between ALDH18A1 and YG1702 with a low Ka. YG1702 not only inhibits ALDH18A1 expression but also leads to the downregulation of MYCN expression, which provides a possible indirect strategy for MYCN targeting. The *in vivo* results obtained for YG1702 have revealed significant inhibition of NB xenografts in animal models of cancer. However, our experiments did not validate the conformational relationship or chemical stability of YG1702, which will be required before its use *in vivo*. More reliable experimental validation of the chemical structure of YG1702, such as *in vitro* testing of YG1702 analogues, is necessary to demonstrate that this compound is indeed a lead drug candidate. The *in vitro* inhibitory activity of this compound against ALDH18A1 should also be quantitatively demonstrated. YG1702 has two ester groups, which typically exhibit low stability and are frequently hydrolysed. Although *in vitro* and *in vivo* experiments have demonstrated the effectiveness of YG1702, some issues remain that need to be further addressed. (1) The solubility of YG1702 is poor and should be increased by cyclodextrin inclusion, microemulsion or liposomes, and micelles. (2) The inhibitory effect of YG1702 on ALDH18A1 enzyme activity needs to be further investigated. (3) The structure of YG1702 should be explored to improve the stability and effectiveness of the inhibitor.

Addressing the following unknowns will help us more fully understand the role of ALDH18A1 in cancers and develop more effective therapeutic measures: (1) as mentioned above, P5CS.long and P5CS.short, which are obtained by exon sliding of the ALDH18A1 gene, are distributed in different tissues. P5CS.long is mainly involved in glutamate biosynthesis, and P5CS.short is involved in the biosynthesis of arginine, which suggests that these two forms of P5CS play different roles in different cancer environments. Therefore, determining whether the expression and function of these two forms of P5CS shift to adapt to the cancer microenvironment is of interest. (2) Because ALDH18A1 is a potential target in cancer therapy, its specific inhibitor must be explored. The crystal structure of P5CS has been deposited in the RCSB protein database (PDB ID: 2H5G). Therefore, molecular docking can be used to develop targeted P5CS inhibitors or ligands; this approach is common in drug design. However, P5CS contains two enzyme structural domains, γ-GK and γ-GPR. Which structural domain is more suitable for designing specific inhibitors remains unclear, and specific compounds based on γ-GK and γ-GPR may exhibit different inhibition efficiencies. (3) Glutamine is transported into the cell by ACST2 and then converted to glutamate by GLS. In addition, glutamate is converted to α-KG, which participates in the TCA cycle or is converted to proline or arginine by ALDH18A1. When the expression or activity of ALDH18A1 is altered, other glutamate-related metabolic enzymes in cancer cells may be regulated to compensate for this effect. Therefore, the relationship between ALDH18A1 and other glutamate-related metabolic enzymes must be investigated. (4) CSCs, also known as TICs, are a small subpopulation of cancer cells that promote tumorigenesis, progression, drug resistance, and recurrence. There is clear evidence showing that some members of the ALDH family, particularly ALDH1A3 and ALDH1A1, are expressed in CSCs and can regulate cellular functions^125^. Various ALDHs play specific biological roles in cancer cells. Because ALDH18A1 is an enzyme involved in amino acid metabolism, its expression level and function in CSCs are unknown and need to be further investigated. In conclusion, understanding the regulatory role of ALDH18A1 in tumorigenesis, progression and drug resistance would be helpful.

## Consent form

All the authors agree with the submission and publication of this manuscript.

## Data Availability

Data sharing is not applicable to this article as no new data were created or analysed in this study.
